# A general framework for fidelity assessment

**DOI:** 10.3389/frhs.2026.1838719

**Published:** 2026-07-29

**Authors:** Dean L. Fixsen, Melissa K. Van Dyke, Karen A. Blase

**Affiliations:** Active Implementation Research Network, Inc., Albuquerque, New Mexico, NM, United States

**Keywords:** essential components, evidence-based, fidelity, measurement, scaling

## Abstract

A general framework for fidelity assessment is outlined to establish common concepts and common measures related to the science of innovations, implementation, and scaling. Fidelity is defined as the extent to which the essential components of an innovation are used in practice. The function of fidelity assessment is to measure the presence and strength of the essential components of an innovation. The general framework identifies the dimensions of fidelity as context, content, and competence, and identifies direct observation, record reviews, and asking others as methods to measure the presence and strength of the essential components. Previous frameworks and uses of fidelity at scale are reviewed. Multifaceted fidelity assessments are used to illustrate the categories in the general framework for fidelity assessment. The general framework can be useful to support quality assurance and sustainable scale-up of interventions, especially for those provided to adversity-affected communities and in low-resource settings.

## Introduction

The interest in evidence-based innovations can be traced to a review of therapy outcome studies by Eysenck (1) who concluded that, “the existence and effectiveness of [therapy] is…unsupported by any scientifically acceptable evidence.” The review was the beginning of decades of research to develop effective innovations and implementation processes (2–5). The quest to produce scientifically acceptable evidence focused attention on the research designs and statistical methods used to generate evidence (6, 7). As improved research methods were used to study outcomes, internal validity (the basic minimum without which any experiment is uninterpretable) and external validity (the question of generalizability) of the innovation (the independent variable) became topics of concern (7). In this context, Yeaton and Sechrest (8) pointed out that consistent monitoring is needed to detect the degree to which treatment is delivered as intended. For an innovation that is not used with integrity “its failure to have any effects becomes totally uninteresting” (p. 161).

As a result of early efforts to define innovations and evaluate outcomes, fidelity of the use of innovations emerged as an important part of the evidence-based movement. Over the years, fidelity has been the subject of systematic reviews (9–18). A recent systematic review of fidelity assessments related to nonspecialists who deliver interventions in lower resource settings concluded that more precise terminology is needed across the implementation and fidelity literatures and greater clarity is needed regarding concepts such as competence and adherence (19).

The goal of this paper is to summarize efforts to define and categorize fidelity and to propose a general framework for fidelity assessment that can serve as a guide for summaries of the literature and for the development of future assessments. Examples of fidelity assessments are provided to illustrate the categories in the general framework.

## Fidelity and innovations

**Figure 1:**
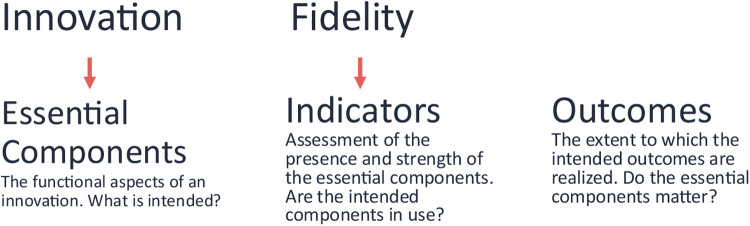
The function of fidelity in science and practice.

Fidelity is defined as the extent to which the essential components of an innovation are used in practice (20). Innovations are used in practice by “intended users” such as therapists, implementation specialists, improvement managers, or others. The fidelity assessment is linked to outcomes. The purpose of evidence-based programs and other innovations is to provide benefits to individuals and populations. Thus, fidelity links the presence and strength of the essential components with beneficial outcomes. These factors are shown in Figure 1. What is intended? Are the intended components assessed? Does doing what is intended matter? Fidelity assessment provides assurance that the innovation is used as intended and that outcomes reasonably can be attributed to the innovation.

Innovations are defined by their essential components. The function of fidelity is to assess the presence and strength of the essential components of an innovation. As noted in Figure 1, the essential components are the aspects of an innovation that have to be there or the innovation is not the innovation. For example, a plan-do-study-act-cycle improvement process has five essential components (PDSAC). The plans vary widely depending on the problem to be solved, cycles may be short-term or occur over many years, and so on. However, it is not an improvement process unless all five components are used as intended (21, 22).

In Figure 1, the innovation refers to human behavior that is interactive and reactive (23). The related literature and the iterative process for establishing the essential components of an innovation have been described (24). The interactive nature of innovations involving human behavior complicates the life of behavioral scientists and practitioners, and it makes fidelity assessment critical for science and practice.

For science, fidelity provides assurance that the procedures described in the methods section were carried out as intended as noted by Mediavilla, García-Vázquez (25) in their experiments with the Doing What Matters in Times of Stress program. This has been referred to as procedural fidelity (26, 27). If the fidelity assessment indicates that the essential components are present, then the outcomes can be related to the intended essential components (28–31). Of course, it is quite possible to have well-established essential components and a good fidelity assessment and also have poor outcomes. For example, Acarturk, Kurt (32) used a well-described group problem solving intervention with Syrian refugees in urban settings. Fidelity was assessed at 78% but depression and anxiety outcomes were not improved.

Even so, the clarity of what was done and what was found provides a useful starting point. As research progresses, the essential components can be revised and refined (33) and the fidelity assessment can be revised and refined (34). As research progresses, an innovation is developed so that the outcomes become more positive and certain. When there is sufficient evidence of the essential components-fidelity-outcomes link, then the innovation is a candidate for scaling in practice.

For practice, as the innovation is replicated and scaled to achieve meaningful benefits for a population, fidelity provides assurance that the essential components of an innovation are present and at sufficient strength to produce beneficial outcomes in each use (35–37). The established essential components-fidelity-outcomes link means that fidelity assessment is the primary measure of scaling success. If fidelity scores are high, then outcomes should be quite good. This is important because fidelity can be known in the present time while outcomes likely will not be realized until some future time.

The importance of fidelity in practice is highlighted when tasks are shifted to non-specialists or when conditions relevant to an innovation are changed in a planned way (19, 38). This may be required in low resource environments or when availability of candidates to fill required roles is limited (39). Fidelity assessment is the sensor to detect divergence from the intended use of the essential components of an innovation. It separates the who and the what: even though *who* is using the innovation to provide services may change in a planned way, *what* needs to be provided still must meet fidelity requirements so that intended outcomes can be achieved.

## Fidelity dimensions and measures

A framework is a mid-level theory (40), a conceptual framework to account for current evidence and to provide testable statements to produce new evidence (41). Current evidence regarding the fidelity dimensions and measures proposed in the general framework is derived from often cited approaches to fidelity assessment and from the dimensions identified in systematic reviews of the literature regarding the measurement and uses of fidelity.

Various frameworks have been proposed to define fidelity dimensions and measures. The first framework for assessing fidelity was described by Hall and Loucks (42) as part of the Concerns-Based Adoption Model (CBAM). The CBAM Innovation Configuration framework is used to operationalize the essential components of an innovation to distinguish “the behaviors of innovation users and nonusers” (p. 265). The CBAM framework continues to be advocated as a way to define (innovation configuration) and assess innovations (43–45).

Subsequent fidelity frameworks continued to focus on the essential components of innovations (46). Waltz, Addis (47) described the dimensions of fidelity as adherence and competence and noted that “competence presupposes adherence, but adherence does not necessarily imply competence” (p. 620). Perepletchikova, Treat (48) used the same adherence-competence dimensions in their review of fidelity in psychotherapy outcome studies. Dane and Schneider (13) further expanded the adherence dimension of fidelity to include exposure, quality of delivery, participant responsiveness, and program differentiation. A systematic review and analysis of 20 fidelity frameworks found “constructs relating to the delivery of an intervention as intended (i.e., adherence, dose, quality) are at the heart of the concept of fidelity” (14).

Mowbray, Holter (49) provided the next advance in fidelity frameworks, adding structure and process as dimensions in addition to the essential components of the innovation. Century, Rudnick (46) also referred to structure as the method for service delivery (e.g., one hour verbal exchange in a clinic) and process as the way the services are delivered (e.g., rehearse new behavior). Fidelity criteria include a) dosage (the length, intensity, and duration of services), b) content, procedures, and activities over the length of the service, c) roles, qualifications and abilities of staff, and d) inclusion/exclusion characteristics for the target population. Carroll, Patterson (50) focused on dosage or exposure (the amount of an intervention received by participants) and adherence (the frequency, duration of the intervention being delivered), and added another dimension named coverage (“whether all the people who should be participating in or receiving the benefits of an intervention actually do so”).

Measurement of the dimensions of fidelity has received attention as well (17). Waltz, Addis (47) encouraged assessment of behaviors that are essential to the treatment as well as behaviors that are proscribed. Mowbray, Holter (49) described fidelity measures that include observations by experts, documentation in program or client records, and interviews or surveys of individual receiving services or closely related to them. Sanetti and Collier-Meek (26) and Bergmann, Niland (34) have described and compared various direct observation and permanent product methods to assess adherence and fidelity. The review conducted by Bond, Simmons (19) found most fidelity assessments include direct observation of performance, even in high stress environments. Schoenwald and Garland (16) conducted a review of the literature and identified 249 unique fidelity measurement methods related to a wide variety of treatment models, recipients, and practitioners. They found that 71.5% of the measures were based on direct observation.

Mowbray, Holter (49) encouraged correlating fidelity and outcomes. Correlating fidelity and outcomes separates form (e.g., frequency, duration, exposure, dosage, content) and function (e.g., credible assessment of essential components that are known and that have a beneficial outcome when used as intended). In their review, Dane and Schneider (13) found that 13 (8%) of 162 outcome studies analyzed the relationship between fidelity and outcomes. Schoenwald and Garland (16) found about 10% of the studies in their review reported fidelity-outcome relationships. In an analysis of fidelity-outcome relationships, Strain (51) “found that providers need to approximate high fidelity before significant changes in child behavior are evident” (p. 110). This is the first suggestion that there may be a curvilinear relationship between fidelity and outcomes, with desired outcomes being achieved only after fidelity is above 80%. Brunk, Chapman (52) found a non-linear pattern for fidelity with the MST Program Performance Index (PPI). An, Dusing (53) suggested setting a predetermined benchmark for fidelity (≥80%) to ensure the independent variable is available prior to conducting an experiment.

In summary, previous frameworks have identified adherence, competence/quality of delivery, structure, and process as dimensions of fidelity, with exposure/dosage/content, quality of delivery, and roles/qualifications of staff as subcategories. Measurement is with direct observation, information from documentation and records, or interviews or surveys of individuals receiving services or closely related to those individuals.

## Considering the dimensions of fidelity

While there is growing clarity about the dimensions that define fidelity, the factors outlined in Figure 1 offer clarity about what fidelity is not. For example, in the literature cited above, Dane and Schneider (13) proposed participant responsiveness and program differentiation as dimensions of fidelity. Carroll, Patterson (50) added a dimension named coverage (“whether all the people who should be participating in or receiving the benefits of an intervention actually do so”). In a review of the literature, Toomey, To (14) identified 20 fidelity frameworks and all the frameworks considered fidelity as a multi-faceted concept comprised of multiple dimensions. The identified dimensions were delivery (e.g., adherence, exposure, and quality of delivery), participants (e.g., receipt of, responsiveness to, and engagement with an intervention), design (e.g., theoretical fidelity, intervention differentiation, and intervention complexity), training (e.g., to facilitate their delivery of the intervention as intended), and context (e.g., recruitment of intervention recipients, organizational characteristics, patient and provider socioeconomic status).

Considering all the information:

1. Participant responsiveness is not an essential component of an innovation. Participant responsiveness may be an innovation outcome. If participant responsiveness is an important outcome of using an innovation, then the essential components related to that outcome can be established during the development of the innovation.
2. Program differentiation is not an essential component of an innovation. Program differentiation is the result of establishing fidelity assessments. Identifying and measuring the essential components of an innovation allows users to differentiate that innovation from any other innovation or from standard practice.
3. Coverage is not an essential component of an innovation. Coverage is the result of establishing the essential components of an innovation then using planned implementation processes to replicate the innovation so that the essential components are available to produce desired outcomes for the intended population.
4. Training is not an essential component of an innovation. Training can be a planned implementation process that is used to put an innovation into effect so that it is used with fidelity.
5. Design is not an essential component of an innovation. Design factors influence the process of establishing the essential components of an innovation so that any requirements related to theory, sufficient complexity to meet a need, and so on are satisfied.

Fidelity is an assessment of the essential components of an innovation and should not be confused with other goals. Other goals are legitimate, and the various related activities may be important for other purposes, just not for assessing the presence and strength of the use of the essential components when an innovation is being used in science or practice.

## Fidelity in everyday use

While frameworks for fidelity assessment were being proposed for outcome studies, evidence-based innovations that had been established, replicated, and scaled were using fidelity assessments on a broad scale. Several examples are described in this section. While the innovations vary widely, in each case the fidelity assessment is linked directly to the unique essential components of the innovation.

For example, the Teaching-Family Model (54, 55) began in 1967 at Achievement Place, a group home for adjudicated youth referred by the juvenile court. Initial attempts to replicate the evidence-based program produced mixed results. A fidelity assessment was developed (56) to discriminate successes from failures and has been in use for over 50 years in every Teaching-Family Site (57, 58). Additional information on fidelity related to this program is presented in later sections of this paper.

Assertive Community Treatment (ACT) is another early example of an evidence-based innovation (59, 60). ACT provides intensive support for adults with serious co-occurring mental health and substance use disorders so that they can function in the community and avoid hospitalization. The Dartmouth Assertive Community Treatment Scale (DACTS) (61) was developed to assess the fidelity of the use of the essential components of ACT. Ongoing use of fidelity assessment is reported by Harrison and Taylor (62) who describe 10 years of DACTS fidelity data for all ACT programs in one state mental health system. DACTS includes 28 items that assess organizational processes, team staffing, and services provided by ACT. Using standard protocols, trained assessors rate information taken from several sources including reports of program behavior from program supervisors or staff; documents reflecting program authority, responsibility, policies, and procedures; management information system or other sources of quantitative data on staffing, clientele, and services. Ratings are made based on a 5-point anchored scale for each of the 28 items. For example, one item rates *in-vivo* Services defined as “Program works to monitor status and develop living skills in community rather than in office.” The anchored rating scale for that item is: Score of 1 = less than 20% time in the community, 2 = 20%–39%, 3 = 40%–59%, 4 = 60%–79%, 5 = 80% of total service time in community. The fidelity assessment items relate to adherence and structure measured by ratings of information obtained from documentation and records and from interviews or surveys of individuals.

Multisystemic therapy® (MST) is an intensive, short-term, homebased treatment program for adjudicated youths who are serious offenders (63, 64). A fidelity assessment called the Therapist Adherence Measure (TAM) was developed (65, 66). The TAM is used in each MST replication as MST has scaled in the US and Europe. TAM includes 26 items that relate to the use of the nine essential components of MST. After a therapist's home visit a designated (non-clinical) staff person conducts a telephone interview with the caregiver (usually a parent) and (separately) with the referred youth. Each individual rates each question on a 5-point scale from 1 (Not at all) to 5 (Very much). The data are entered into the MST online data collection and reporting system. The benchmark for MST fidelity is 16/26 items scored as 5 (Very Much). Sample questions are: “My family knows exactly which problems we were working on.” “The therapist tried to change some ways that family members interact with each other.” “The therapist understood what is good about our family.” The TAM also includes questions about proscribed practices: “The therapy session included a lot of irrelevant small talk (chit-chat).” “There were awkward silences and pauses during the session.” The fidelity assessment items relate to competence measured by ratings from those who have directly interacted with a practitioner.

Positive Behavior Interventions and Supports (PBIS) is a whole school intervention to reduce discipline problems and improve academic achievement (67). A fidelity assessment called the School-wide Evaluation Tool (SET) was developed to assess the use of the essential components of PBIS (68). The data are entered into the PBIS online data collection and reporting system. Ongoing use of fidelity assessment is reported by McIntosh, Mercer (36) who conducted an analysis of annual SET fidelity data from 5,331 schools for 5 years. SET includes 28 items organized into 7 subscales, each subscale relating to an essential component of PBIS. A designated staff person in a school collects the information and enters the fidelity data (along with student data) into the PBIS national data system where scores are analyzed, graphed, and available to each reporting school. Data are collected through a review of documents, observations, and interviews or surveys of staff and students. The items are scored on a 3-point anchored scale (0 = not in place, 1 = partially in place, 2 = fully in place). The benchmark for fidelity is an overall score of 80%. Sample questions are: “Is there documentation that staff has agreed to 5 or fewer positively stated school rules/ behavioral expectations?” (0 = no; 1 = too many/negatively focused; 2 = yes). “Are the agreed upon rules & expectations publicly posted in 8 of 10 locations?” (0 = 0–4; 1 = 5–7; 2 = 8–10). “Do 90% of staff asked agree with administration on what problems are office-managed and what problems are classroom–managed?” (0 = 0%–50%; 1 = 51%–89%; 2 = 90%–100%). “Can 90% of staff asked report that there is a school-wide team established to address behavior support systems in the school?” (0 = 0%–50%; 1 = 51%–89%; 2 = 90%–100%). The fidelity assessment items relate to adherence and process measured by ratings of information obtained from documentation and from interviews or surveys of individuals.

The Parent Management Training-Oregon™ (PMTO) program is a family-based parent training intervention for children/youth 2–18 years of age with disruptive and anti-social behaviors (69). A fidelity assessment called the FIMP (Fidelity of Implementation) was developed (70). The FIMP is used in each PMTO replication and has been scaled in Michigan (71, 72) and nationally in Norway where fidelity, implementation processes, and outcomes were continually assessed for more than 10 years (73–76). The coded items relate to the five essential components of PMTO intervention. Coding is based on direct observation of videotaped intervention sessions. Therapist behavior is scored on a 9-point scale where 1–3 is “needs work,” 4–6 is “acceptable,” and 7–9 is “good work.” The benchmark for fidelity is a score of 7–9 (good work). Reliable coding requires about 40 h of training for fidelity assessors. Therapist behavior that is observed and coded on the videotapes includes: Demonstrates integration of PMTO tools. Manages orderly flow. Assesses skills and fills in gaps. Prevents/manages conflict. Overall accomplishment of goals. The fidelity assessment items relate to competence measured by direct observation of practitioner behavior by trained assessors.

Active Implementation Specialists™ conduct coaching for competence, an implementation innovation (77). Coaching for competence is based on direct observation of practitioners using an interaction-based program (78, 79). Newly trained practitioners may falter with the words to say and things to do and often do not recognize the cues (good judgement) to use (or not use) parts of the program. Errors of omission are difficult to detect without direct observation of the practitioner and the recipient interacting with one another. Direct observation also provides information on the quality components (tone of voice, proximity, respectful language, body movements, use of humor) and other nuances that may influence outcomes (80). Implementation Teams use the best practices for coaching to assess fidelity of coaching behavior. Two Implementation Team members directly observe the coach and review relevant records. Each item is scored as fully in place, partially in place, or not in place. The items relate to coaching frequency, documentation, and meetings. Expected frequency of coaching is set in advance and is adjusted as practitioners gain experience and grow in their skill competency (e.g., pre-fidelity, after meeting fidelity criteria twice). Required documentation (e.g., written feedback after each coaching visit) and the timeline for submission by the coach (to whom, by when) is specified. Within 24 h of a direct observation the coach meets with the practitioner, provides a verbal summary of the practitioner's use of the essential components, and engages in practice to criterion of one or two skills that need improvement. A brief written summary of observations and recommendations is provided to the practitioner within five working days. The fidelity assessment items relate to adherence information obtained from documentation and competence measured by direct observation of coach's behavior.

In summary, in these diverse examples fidelity is linked to the essential components of each of these innovations and is sufficiently practical to be assessed continually in replications and scaled use over extended periods of time. While not reviewed here, in each case the fidelity scores have been correlated with intended outcomes to establish the essential components-fidelity-outcomes relationship.

## A general framework for fidelity assessment

Previous frameworks and the use of fidelity assessments in standard practice provide a firm foundation for a general framework for fidelity assessment. Given the critical role of fidelity in science, implementation, and scaling (20, 23, 81–83), this paper outlines a general framework for fidelity assessment.

Fidelity is defined as the extent to which the essential components of an innovation are used in practice (20, 24). The function of fidelity assessment is to ensure the presence and strength of the essential components of an innovation. The test of a fidelity assessment is the relationship between fidelity and outcomes (84). A strong correlation (±0.70) between fidelity scores and outcome scores indicates the essential components have been sufficiently identified, adequately measured, and meaningfully related to desired outcomes (24, 85).

The general framework for fidelity assessment provides a universal way to categorize existing fidelity assessments and a guide for developing new assessments. It is intended to clarify “what is fidelity” and “how to measure fidelity” and to establish a common set of concepts to integrate views of fidelity. In recent years, innovation fidelity has been discriminated from implementation fidelity, where implementation fidelity refers to the use of the essential components of an implementation process such as training and coaching processes for practitioner competency development, or processes to create readiness for leaders to engage in organization change so that programs can be used effectively. In the general framework for fidelity assessment, fidelity dimensions and measures apply equally to innovation fidelity and implementation fidelity. Use of the common concepts and common categories of measures are intended to facilitate future reviews and advances pertaining to fidelity, implementation, and scaling.

As outlined in Table 1, in the general framework for fidelity assessment there are three dimensions of fidelity (context, content, and competence) and three ways to assess those dimensions (direct observation, record review, ask others).

**Table 1:** Fidelity assessment dimensions and measures. Used with permission (77).

Dimensions of Fidelity Assessment	Measurement Methods – Data Sources		
	Direct Observation (face-to-face, video, audio)	Record Reviews (electronic or paper documents)	Ask Others (opinions, experiences, observations)
**Context** Prerequisite conditions that need to be in place regarding setting, qualifications, preparation	***Observation of*** the physical setting, timing, scheduling	***Permanent products*** that record time, setting, timing, schedules, record of completion of activities	***Interviews or surveys*** asking about orientation activities, availability of materials ahead of events, perception of preparation of a practitioner
**Content** Extent to which the essential components are used, referenced, monitored, or accessed and may include documenting the absence of activity or content that is proscribed or should be avoided	***Observation of*** use of the essential components or references to the content in discussions, sessions, meetings	***Permanent products*** that reflect the use of the essential components (e.g., plans, meeting minutes, agendas, notes, records)	***Interviews or surveys*** asking about use of curriculum or content; ratings by recipients regarding use of materials, use of instructional practices; ratings by practitioners about critical supports
**Competence** Extent to which the competencies related to the essential components are used skillfully in interactions with others	***Observation of*** ability to engage recipients with information and questions; use of prompt and accurate feedback related to the essential components or processes	***Permanent products*** that demonstrate skillful use of essential components, written feedback that demonstrates deep understanding of the essential components	***Interviews or surveys*** regarding the quality, helpfulness, specificity, timeliness of feedback and treatment provided by practitioners

## Dimensions of fidelity

- Context: Context relates to establishing the conditions under which an innovation is able to be used. This includes prerequisite conditions that need to be in place regarding setting, qualifications, or preparation so that the essential components can be used. For example, availability of equipment, location of services provided, timing, scheduling, or staff qualifications may relate to the presence or use of the essential components and be part of a fidelity assessment.
- Content: Content relates to ensuring the presence of an innovation. This includes the extent to which the essential components are used, referenced, monitored, or accessed and may include assessing the absence of activity or content that is proscribed or should be avoided. For example, indicators of using the procedures related to the essential components, attention to prescribed topics, sequencing of activities, and participation by certain people may be part of a fidelity assessment.
- Competence: Competence relates to the skillful use of an innovation. This includes the extent to which the competencies related to the essential components are used skillfully in interactions with others and may include indicators of the tone of voice or judgement or nuanced uses of essential components.

## Measures of fidelity

- Direct Observation: Practitioner interactions with others can be observed with the fidelity assessor present (face-to-face) or using recordings of live interactions (video or audio) to detect and record information related to the use of the essential components of an innovation.
- Record Reviews: Information related to the use of essential components of an innovation can be available in some records (electronic or paper documents) such as treatment plans, progress notes, checklists, personnel records, and so on.
- Ask Others: Practitioner interactions can be assessed by interviews or surveys of opinions or experiences of those who directly interact with a practitioner and can reflect their personal experience and knowledge related to the practitioner's use of the essential components of an innovation.

The context dimension may include items that assess aspects related to the use of an innovation but not the essential components themselves. This was referred to as structure in other fidelity frameworks. For example, air quality and cleanliness may be assessed as part of a fidelity assessment related to a surgical procedure (86). They may not be essential components of the innovation as performed by a surgeon but they enable or facilitate the benefits of a surgical innovation as it is used.

The content dimension is related to what has been referred to as adherence or procedural fidelity where components of an innovation are scored as present or absent. For example, fidelity assessed the practitioners’ use of the instructional components of discrete-trial instruction (e.g., “present materials as written,” “provide praise contingent on correct response,” “record data before presenting the next trial”). The fidelity assessors scored the occurrence–nonoccurrence of each instructional component as a practitioner interacted with a child with autism (34).

The competence dimension goes beyond content and assesses the quality of practitioner behavior as it relates to using the essential components. For example, the competence of the use of motivational interviewing (MI) in clinical treatment for addictions was assessed (87). Fidelity assessment focused on 10 MI consistent items (those prescribed in MI) and 10 MI inconsistent items involving interventions antithetical to MI (e.g., unsolicited advice). Observers rated each MI consistent item for competence (skill of delivery, with 1 = very poor to 7 = excellent). Competence ratings were anchored with guidelines that reflected higher and lower levels of skill as the therapists performed each item. “Competence presupposes adherence, but adherence does not necessarily imply competence” (47).

The reliability of fidelity measures is assessed with interobserver agreement as two assessors simultaneously and independently observe and rate live observations, extract information from records, and conduct interviews or surveys (88, 89). The goal is to achieve an 80% benchmark for interobserver agreement. The validity of fidelity measures in assessed by relating fidelity scores with outcome scores. The goal is to achieve a benchmark correlation of ±0.70 between fidelity and outcomes.

For record reviews, the linkages between practitioner behavior and the record need to be established to ensure the record is a reasonable and reliable indicator of the presence, absence, or strength of actual practitioner behavior. For example, the record of a treatment visit may have a checkmark next to “capitalized on five or more teaching opportunities when using role play” or a record of staffing may indicate “the practitioner met 80% of all qualifications at the end of training.” The validity of the records must be established sufficiently prior to using the records as data sources for fidelity assessments.

For asking others, the validity of the information is assumed. If a parent rates the practitioner one way or the other, then it is assumed that the parent's rating is an accurate reflection of the parent's experience with the practitioner. Given this assumption, a fidelity assessment specifies who should be asked to ensure that each person being asked has sufficient exposure to the practitioner to make an informed judgement. For example, a fidelity assessor may interview each parent who was present during a therapy session or survey each teacher who is in direct contact with a practitioner at least once each week.

The dimensions and measurement methods are outlined in the 3X3 matrix in Table 1. For the fidelity frameworks reviewed in the prior section, structure, quality of delivery, and roles or qualifications of staff are Context dimensions. Adherence, process, exposure, dosage, and content are Content dimensions. And competence and quality of delivery are Competence dimensions. The data sources suggested by Mowbray, Holter (49) are used in the general framework shown in Table 1. Note that self-report typically is unreliable (81, 90) and is not included as a data source.

The general framework for fidelity assessment provides a way to categorize existing fidelity assessments. In Table 2, the fidelity assessments in everyday use referenced in a previous section are used to illustrate the categories in the fidelity matrix. Assertive Community Treatment (ACT) is based on a team of 10 qualified professionals (e.g., nurse, substance abuse specialist, vocational specialist, psychiatrist) who engage and support up to 100 adults with severe mental health and substance abuse problems who live in the community. The Dartmouth Assertive Community Treatment Scale (DACTS) items assess the Context in which treatment is provided based on evidence of staffing ratios, location of services, and the kinds of services.

**Table 2:** Existing fidelity assessments located in a general framework for fidelity assessment. .

Dimensions of Fidelity Assessment	Measurement Methods – Data Sources		
	Direct Observation (face-to-face, video, audio)	Record Reviews (electronic or paper documents)	Ask Others (opinions, experiences, observations)
**Context** Teague, Bond (61) Dartmouth Assertive Community Treatment Scale (DACTS)		Client/provider ratio of 10:1 High number of service contacts, as needed Takes clients in at a low rate to maintain a stable service environment	Supervisor of front-line clinicians provides direct services Program provides support/skills for client's support network: family, landlords, employers
**Content** Horner, Todd (68) School-wide Evaluation Tool (SET)	Are the agreed upon rules & expectations publicly posted in 8 of 10 locations?	Is there a documented system for teaching behavioral expectations to students on an annual basis? Is the administrator an active member of the school-wide behavior support team?	Can the administrator clearly define a system for collecting & summarizing discipline referrals (computer software, data entry time)? Can at least 70% of 15 or more students state 67% of the school rules?
**Competence** Direct observation Forgatch, Patterson (70) Fidelity of Implementation (FIMP) Ask others Schoenwald, Henggeler (66) Therapist Adherence Measure (TAM)	Prevents/manages resistance Prevents/manages conflict Maintains balance Promotes united approach Focuses on encouragement/support Connects with storyline Sets up, conducts, debriefs, and capitalizes on opportunities when using role play Uses a variety of tools (e.g., humor, reflects, paradox, metaphors)		The therapist tried to understand how my family's problems all fit together. The therapist recommended that family members do specific things to solve our problems. The therapist's recommendations required family members to work on our problems almost every day. The therapist tried to change some ways that family members interact with each other.

Positive Behavior Interventions and Supports (PBIS) is a whole school intervention and the School-wide Evaluation Tool (SET) items assess the collective behavior of staff who generate school-wide expectations for behavior, systematically teach those expectations to students, and constructively deal with exceptions. Fidelity assessment is based on evidence of the Content of PBIS found in school records or by asking students and staff or by looking for posters in high-use locations (e.g., cafeteria).

The Parent Management Training-Oregon™ (PMTO) therapist records every session for Direct Observation of Competence. A fidelity assessor analyzes the video recording of therapist and family interactions to assess a therapist's use of the PMTO essential skills and nuances in response to the ongoing behavior of family members. Multisystemic therapy® (MST) is an intensive, short-term, homebased treatment program. A fidelity assessor Asks Others about Competence. After a home visit, a fidelity assessor contacts parents and the referred youth to rate the therapist's behavior as they experienced it during the home visit.

As noted previously, these fidelity assessments have been in use for many years in thousands of replications of the various evidence-based innovations. The fidelity items, measurement methods, preparation of fidelity assessors, and shared data systems provide a sound starting point for the development of any new fidelity assessment.

## Fidelity assessment example: goals, essential components, skills

The relationships among the goals, essential components, and fidelity assessment methods for an evidence-based program are illustrated in this section.

A multifaceted fidelity assessment was developed for the Teaching-Family Model and has been in use in every Teaching-Family organization for over 50 years (57, 58). The goals of the Teaching-Family program are to provide treatment that is humane, effective, individualized, satisfactory to consumers, cost efficient, and replicable. The essential components were identified and operationalized in usability testing from its inception in 1967 through 64 attempted replications by 1975. The essential components of treatment are relationship development, teaching appropriate alternative behavior, motivation systems. self-determination, and counseling. Clinical judgement also is an essential component with a focus on morals (right, wrong), ethics (good, bad), and judgement (good sense) as Teaching-Family practitioners use the treatment components in their interactions with others. The essential components have been operationalized and the skills are taught in training and coaching and assessed regularly (24, 91). Multifaceted fidelity assessments are especially important in high risk circumstances, for example, when care is provided to dependent youths who are involuntarily placed in a relatively private residential treatment setting (58).

Fidelity assessment for the Teaching-Family Model was designed to assess the presence and strength of the essential components of the treatment program as they were used by Teaching-Parents (Teaching-Family practitioners). The complexity of the essential components of the Teaching-Family Model requires direct observation to detect their use or misuse, and to assess the nuances related to judgement (e.g., when and with whom to use/not use skills; missed opportunities to use skills). In addition, it is not enough to change youth behavior in the group home. Asking others provides an indication of the impact on (generalization of) youth behavior at home, at school, and in the community.

As noted in Table 3, the fidelity assessment relates directly to the essential components of the Teaching-Family treatment program. The fidelity assessment also relates to the goals of the Teaching-Family program to provide treatment that is:

1. Humane: The youths along with their parents and teachers and referral agents are in frequent contact with the Teaching-Parents and the youths and have firsthand knowledge of how the Teaching-Parents interact with the youths. Any strengths, perceptions of mistreatment, or concerns can be reported during the interviews and surveys that are a part of each fidelity assessment (at least annually). The quality dimensions of teaching interactions (i.e., use a calm, caring speaking voice; be calm and matter of fact when offering corrective feedback; make polite and pleasant requests) are observed and rated during the 2 to 3 h in-home visit by the fidelity assessors.
2. Effective: The youths live with the Teaching-Parents and their parents and teachers are in frequent contact with the Teaching-Parents. They have firsthand knowledge of how the youths are progressing at home, at school, and in the community. In addition to “asking others” the two assessors have many opportunities during the 2 to 3 h in-home visit to observe and rate the appropriateness of the Teaching-Parents use of the essential components of the Teaching-Family treatment program to support behavior change.
3. Individualized: Throughout the 2 to 3 h in-home visit, the two assessors observe and rate the appropriateness of the Teaching-Parents’ interactions with each youth. This also is rated as part of the record review of individual youth files.
4. Satisfactory to consumers: The youths and their parents, teachers, and referral agents are asked directly, “How satisfied are you that…?”

**Table 3:** Teaching-Family model multifaceted fidelity assessment categorized.

Dimensions of Fidelity Assessment	Measurement Methods – Data Sources		
	Direct Observation (face-to-face, video, audio)	Record Reviews (electronic or paper documents)	Ask Others (opinions, experiences, observations)
**Context** Prerequisite conditions that need to be in place regarding setting, qualifications, preparation	Condition of the home (organization responsibility) is assessed by noting the physical condition of the group home during the in-home visit by two assessors.		
**Content** Extent to which the essential components are used, referenced, monitored, or accessed and may include documenting the absence of activity or content that is proscribed or should be avoided		Record keeping is assessed by reviewing the completeness and content of the file for each youth in residence with items related to referral reasons, individual treatment plans, and progress notes.	
**Competence** Extent to which the competencies related to the essential components are used skillfully in interactions with others	Two assessors use standard protocols during a 2–3 h visit to rate and comment on the essential components of treatment (relationship development, teaching appropriate alternative behavior, motivation systems. self-determination). Assessors note commissions (what was done) and omissions (what should have been done but was not done when the opportunity arose).		During the in-home visit, each youth is interviewed privately and asked to rate and comment on Teaching-Parent fairness, concern, helpfulness, etc. Within 10 days of the in-home visit, survey questions are sent to each parent, teacher, referral/funding agent, etc. who has direct contact with the youths and the Teaching-Parents, and are asked to rate and comment on correcting problems, cooperation, communication, and effectiveness.

The goals of being cost efficient and replicable are not part of the fidelity assessment since they are not within the purview of individual Teaching-Parents. Independent analyses have established cost efficiency (92, 93) and ability to replicate the Teaching-Family Model (57, 94, 95).

Each fidelity assessment item asks, “How satisfied are you that…?” For the youths, parents, teachers, and others in the “asking others” column of Table 3, “satisfaction” is an undefined term. It is up to each individual to decide whether he or she is satisfied or not according to his or her own experiences and perceptions. If one person is less than satisfied, then that rating and his or her comments are taken seriously no matter how others might judge the reasoning.

For fidelity assessors, “satisfaction” is defined and observations are calibrated during the development of assessors and checked again by calculating interobserver agreement between the two assessors who conduct an in-home visit (77). During the development of assessors and periodically thereafter, the accuracy and quality of the comments accompanying a satisfaction rating also are the subject of discussion and agreement. This is important because the comments made by assessors are used to inform the Teaching-Family practitioner and coach so that excellent use of the essential components is reinforced with examples, and specific areas for improvement are noted with rationales.

As illustrated by the Teaching-Family example, fidelity assessment items relate directly to the skills needed to carry out the essential components of the innovation (e.g., relationship development, teaching appropriate alternative behavior) and can be summarized to assess the broader goals of the innovation as well (e.g., humane, effective, individualized). Components of the multifaceted Teaching-Family fidelity assessment fit the categories of the general framework and illustrate the use of the general framework.

## Conclusion

Fidelity assessment is not a luxury or a burden. Fidelity is required in research to establish the evidence for the presence and strength of the independent variable (the innovation) so that outcomes can be related to the intended essential components described in a methods section. In subsequent research, fidelity is the standard to meet when attempting to replicate and scale an innovation. Fidelity assessment ensures the essential components are present and strong enough to produce expected outcomes in each use of an innovation.

The general framework for fidelity assessment reflects the accumulation of knowledge over the past several decades and clarifies the dimensions of fidelity and the measures of fidelity. Each dimension and each measurement relates to the essential components of the innovation. The 3X3 matrix provides common concepts and common language to enhance the value of systematic reviews of the literature and inform the development of new fidelity assessments.

Science is based on the accumulation of knowledge and knowledge is based on facts (96, 97). With common concepts, common language, and common measures, the science of innovations, implementation, and scaling can crowdsource the development and systematic accumulation of knowledge. The general framework for fidelity assessment is a step down the science to service path.

## Limitations

The general framework includes predictions and testable statements that may give the appearance of certainty where the evidence is not yet available. The general framework describes categories of dimensions and measures of fidelity. The definitions of categories in the framework do not explicate alternative views or tradeoffs among the views. The measurement categories are not explained in terms of validity or reliability. Finally, the dimensions, measures, and concepts in the general framework are oriented to developing and scaling innovations to achieve socially significant benefits for individuals and populations. Concepts oriented to basic research may require different views of fidelity, its measurement, and its meaning.

## Data Availability

The original contributions presented in the study are included in the article/Supplementary Material, further inquiries can be directed to the corresponding author.
